# Mapping SARS-CoV-2 Antibody Epitopes in COVID-19 Patients with a Multi-Coronavirus Protein Microarray

**DOI:** 10.1128/Spectrum.01416-21

**Published:** 2021-10-27

**Authors:** David Camerini, Arlo Z. Randall, Krista Trappl-Kimmons, Amit Oberai, Christopher Hung, Joshua Edgar, Adam Shandling, Vu Huynh, Andy A. Teng, Gary Hermanson, Jozelyn V. Pablo, Megan M. Stumpf, Sandra N. Lester, Jennifer Harcourt, Azaibi Tamin, Mohammed Rasheed, Natalie J. Thornburg, Panayampalli S. Satheshkumar, Xiaowu Liang, Richard B. Kennedy, Angela Yee, Michael Townsend, Joseph J. Campo

**Affiliations:** a Antigen Discovery Incorporated (ADI), Irvine, California, USA; b University of California, Irvine, California, USA; c Centers for Disease Control and Prevention, Atlanta, Georgia, USA; d Mayo Clinic, Rochester, Minnesota, USA; University of Sussex

**Keywords:** COVID-19, HCoV, SARS-CoV-2, antibody binding sites

## Abstract

The rapid worldwide spread of SARS-CoV-2 has accelerated research and development for controlling the COVID-19 pandemic. A multi-coronavirus protein microarray was created containing full-length proteins, overlapping protein fragments of various lengths, and peptide libraries from SARS-CoV-2 and four other human coronaviruses. Sera from confirmed COVID-19 patients as well as unexposed individuals were applied to multicoronavirus arrays to identify specific antibody reactivity. High-level IgG, IgM, and IgA reactivity to structural proteins S, M, and N of SARS-CoV-2, as well as accessory proteins such as ORF3a and ORF7a, were observed that were specific to COVID-19 patients. Antibody reactivity against overlapping 100-, 50-, and 30-amino acid fragments of SARS-CoV-2 proteins was used to identify antigenic regions. Numerous proteins of SARS-CoV, Middle East respiratory syndrome coronavirus (MERS-CoV), and the endemic human coronaviruses HCoV-NL63 and HCoV-OC43 were also more reactive with IgG, IgM, and IgA in COVID-19 patient sera than in unexposed control sera, providing further evidence of immunologic cross-reactivity between these viruses. Whereas unexposed individuals had minimal reactivity against SARS-CoV-2 proteins that poorly correlated with reactivity against HCoV-NL63 and HCoV-OC43 S2 and N proteins, COVID-19 patient sera had higher correlation between SARS-CoV-2 and HCoV responses, suggesting that *de novo* antibodies against SARS-CoV-2 cross-react with HCoV epitopes. Array responses were compared with validated spike protein-specific IgG enzyme-linked immunosorbent assays (ELISAs), showing agreement between orthologous methods. SARS-CoV-2 microneutralization titers were low in the COVID-19 patient sera but correlated with array responses against S and N proteins. The multi-coronavirus protein microarray is a useful tool for mapping antibody reactivity in COVID-19 patients.

**IMPORTANCE** With novel mutant SARS-CoV-2 variants of concern on the rise, knowledge of immune specificities against SARS-CoV-2 proteins is increasingly important for understanding the impact of structural changes in antibody-reactive protein epitopes on naturally acquired and vaccine-induced immunity, as well as broader topics of cross-reactivity and viral evolution. A multi-coronavirus protein microarray used to map the binding of COVID-19 patient antibodies to SARS-CoV-2 proteins and protein fragments as well as to the proteins of four other coronaviruses that infect humans has shown specific regions of SARS-CoV-2 proteins that are highly reactive with patient antibodies and revealed cross-reactivity of these antibodies with other human coronaviruses. These data and the multi-coronavirus protein microarray tool will help guide further studies of the antibody response to COVID-19 and to vaccination against this worldwide pandemic.

## INTRODUCTION

A novel human coronavirus which causes severe acute respiratory syndrome, now known as SARS-CoV-2, emerged in December 2019. Infection with SARS-CoV-2 spread rapidly worldwide, and on 11 March 2020, it was declared a pandemic by the World Health Organization (WHO) (1). As of 7 May 2021, there are over 156 million confirmed cases of coronavirus disease (COVID-19) caused by this new virus, resulting in more than 3.2 million deaths, corresponding to a case mortality rate of ∼2.1% (https://coronavirus.jhu.edu/). Best estimates indicate that SARS-CoV-2 has a basic reproductive number, *R*_0_, of 2 to 2.5 and an incubation time of approximately 4.6 days (2, 3), which allows rapid spread of the virus. Diagnosis, treatment, and vaccination against COVID-19 will all benefit from a clear understanding of the immune response to SARS-CoV-2 infection.

Previous studies have shown that COVID-19 patients rapidly seroconvert to SARS-CoV-2 and produce IgM, IgG, and IgA antibodies directed to several viral proteins (4–7). Reinfection challenge studies in rhesus macaques showed that the humoral and cellular immune response to SARS-CoV-2 infection was effective in blocking reinfection (8, 9). Nevertheless, it is not clear whether all antibody responses are beneficial or whether some antibody responses to SARS-CoV-2 lead to a less favorable course of disease (10, 11). Moreover, enhancement of infection by antibodies has been reported for severe acute respiratory syndrome coronavirus (SARS-CoV), which is closely related to SARS-CoV-2 (12–15).

We have created and used a multi-coronavirus protein microarray containing over 900 coronavirus proteins, protein fragments, and peptides to map IgG, IgA, and IgM antibody epitopes in sera from COVID-19 patients. Our approach localizes the antibody reactivity of COVID-19 patients within SARS-CoV-2 proteins and allows us to map the bound antigenic regions. Furthermore, we can similarly measure the antibody reactivity of COVID-19 patients and healthy controls with endemic human coronaviruses and with the two previous epidemic coronaviruses, SARS-CoV and Middle East respiratory syndrome coronavirus (MERS-CoV). Our findings and the multi-coronavirus protein microarray we created will be useful in discerning which *de novo* and cross-reactive antibody responses to SARS-CoV-2 are protective and which may be less useful in preventing disease or may even be detrimental. In addition, if high levels of antibody to specific epitopes are found to be especially protective, the array could be used to screen convalescent plasma for therapeutic potential and vaccine recipient sera as a preliminary measure of efficacy (16–19).

## RESULTS

The multi-coronavirus protein microarray created and used in this study encompasses over 900 features. It includes the 4 structural proteins and 5 accessory proteins of SARS-CoV-2 as well as overlapping 100-, 50-, and 30-amino acid (aa) protein fragments to map immunodominant domains within each of these 9 SARS-CoV-2 proteins. It also contains the structural proteins of SARS-CoV, MERS-CoV, HCoV-NL63, and HCoV-OC43, plus overlapping 13- to 20-aa peptides of the SARS-CoV structural proteins and of the S proteins of MERS-CoV, HCoV-NL63, and HCoV-OC43 (Table 1).

**TABLE 1:** Features of the 1st-generation ADI multi-coronavirus protein microarray^*a*^

Virus	S protein	E protein	M protein	N protein	Accessory or other proteins	No. of features (total *n* = 934)
SARS-CoV-2	RBD^*b*^, S^*c*^ from BEI, S1, S2 and 30-, 50-, and 100-aa fragments by IVTT at ADI	Protein and 30- and 50-aa fragments by IVTT at ADI	Protein and 30-, 50-, and 100-aa fragments by IVTT at ADI	Protein and 30-, 50-, and 100-aa fragments by IVTT at ADI	ORF 3a, 6, 7a, 8, and 10 protein and 30-, 50-, and 100-aa fragments by IVTT at ADI	12 proteins, 310 fragments = 322 features
SARS-CoV	Protein^*d*^ and peptide set from BEI Resources	IVTT by ADI, peptides from BEI Resources	Protein, peptides from BEI Resources	Protein, peptides from BEI	3CL protease from BEI Resources	5 proteins, 265 peptides = 270 features
MERS-CoV	Protein from BEI Resources	Protein produced by IVTT at ADI	Protein produced by IVTT at ADI	BEI Resources	ORF 3a, 4a, 4b, 5, and 8b proteins produced by IVTT at ADI	9 proteins
HCoV-NL63	Protein by IVTT at ADI, peptides from BEI	Protein produced by IVTT at ADI	Protein produced by IVTT at ADI	Protein produced by IVTT at ADI	ORF 3 protein produced by IVTT at ADI	5 proteins and 96 peptides = 101 features
HCoV-OC43	Protein by IVTT at ADI, peptides from BEI	Protein produced by IVTT at ADI	Protein produced by IVTT at ADI	Protein produced by IVTT at ADI	HE, N2 protein IVTT at ADI	6 proteins and 226 peptides = 232 features

a IVTT means coupled *in vitro* transcription and translation.

b RBD is the receptor binding domain, aa 319 to 541 of the SARS-CoV-2 S protein.

c SARS-CoV-2 S protein is a stabilized form with a trimerization sequence and transmembrane domain deletion.

d SARS-CoV-S protein is a transmembrane domain deleted form.

The multicoronavirus array was incubated with sera from two sets of COVID-19 patient samples and associated negative controls collected in different regions of the United States. The first set of sera from 10 COVID-19 patients and 10 prepandemic healthy donors was obtained from the Centers for Disease Control and Prevention (CDC) in Atlanta, Georgia. The second set included sera from 10 COVID-19 patients and 9 prepandemic samples obtained from the Mayo Clinic in Rochester, Minnesota. The age, sex, and SARS-CoV-2 enzyme-linked immunosorbent assay (ELISA) results of the COVID-19 patients and healthy control blood donors in both sample sets are shown in Tables 2 and 3. ELISAs were performed separately at the two different sites. Both assays clearly discriminated the COVID-19 patient samples from the control samples.

**TABLE 2:** Serum donors for samples obtained from the CDC

Sample ID^*a*^	SARS-CoV-2 S ELISA (S/T)^*b*^	Age (decade)	Sex
N1	0.25	60s	M
N2	0.09	20s	M
N3	0.18	40s	M
N4	0.34	50s	M
N5	019	60s	M
N6	0.94	40s	M
N7	0.26	40s	M
N8	0.15	50s	M
N9	0.40	40s	M
N10	0.19	50s	M
P1	2.06	60s	F
P2	3.98	60s	M
P3	5.47	50s	F
P4	2.35	20s	F
P4	5.16	70s	F
P6	5.21	70s	M
P7	3.82	30s	M
P8	5.30	80s	M
P9	3.81	40s	F
P10	5.20	40s	M

a N, healthy negative control; P, COVID-19-positive patient.

b S/T, signal/threshold for positivity ratio; >1.0 is positive.

**TABLE 3:** Serum donors for samples obtained from the Mayo Clinic

Sample ID^*a*^	SARS-CoV-2 S ELISA (S/C)^*b*^	Age (decade)	Sex
N101	<0.4	10s	M
N102	<0.4	10s	F
N103	NA	10s	M
N104	<0.4	10s	M
N105	NA	10s	M
N106	NA	10s	M
N107	<0.4	10s	F
N108	NA	10s	F
N109	<0.4	20s	F
P101	2.76	50s	F
P102	3.71	50s	M
P103	3.83	70s	M
P104	1.42	30s	M
P105	4.24	10s	F
P106	2.48	40s	F
P107	1.5	50s	F
P108	3.05	60s	F
P109	2.03	70s	M
P110	3.6	70s	M

a N, healthy negative control; P, COVID-19-positive patient.

b S/C, signal/calibrator ratio; >1.1 is positive. NA, not assayed, but collected prior to November 2019.

### Specific antibody reactivity to SARS-CoV-2 and SARS-CoV purified recombinant proteins in COVID-19 patients.

**FIG 1:**
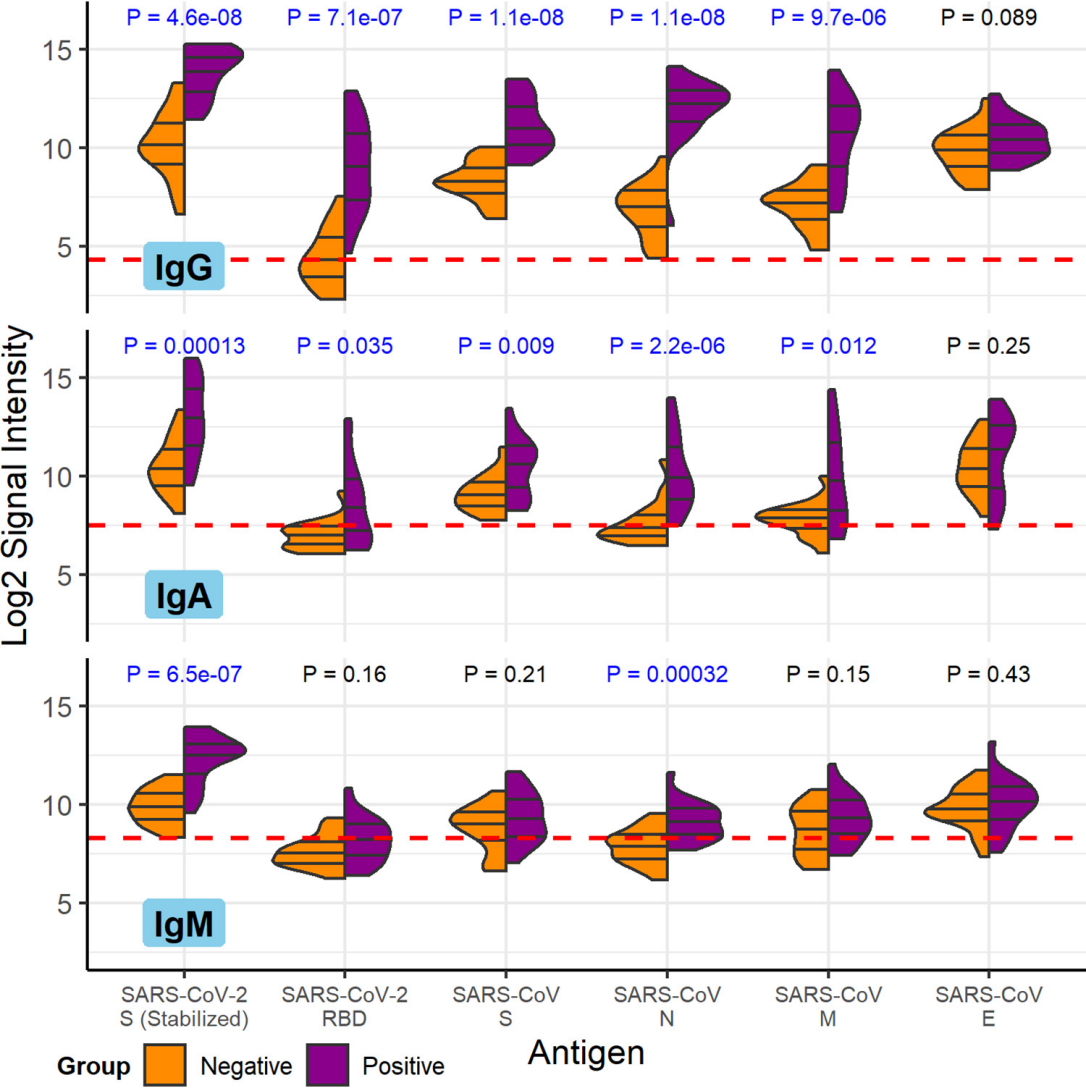
COVID-19 patient and healthy control antibody reactivity with purified SARS-CoV-2 and SARS-CoV proteins. The split violin plot shows the log_2_-transformed fluorescence signal intensity distribution of antibodies bound to each purified protein on the multi-coronavirus protein microarray. Within each half-violin are three lines representing the interquartile range and the median. Above each split violin is the Wilcoxon rank sum *P* value, colored blue for significant *P* values below 0.05. The three panels are split by isotype (IgG, top; IgA, middle; IgM, bottom). Horizontal red dashed lines are drawn at the median of all signal intensities against purified proteins (*n* = 14) and peptides (*n* = 587) plus 1.0, i.e., double the global median; this threshold serves as a point of reference but not necessarily a seropositivity cutoff for each protein.

The specimens from COVID-19 patients had robust anti-SARS-CoV-2 IgG and IgA antibodies. IgM antibody responses were weaker. The magnitude and specificity of antibody responses were similar in the samples obtained from the CDC and the Mayo Clinic, so they are presented together here. COVID-19 patient serum IgG, IgA, and IgM reacted strongly to purified SARS-CoV-2 spike (S) protein as well as SARS-CoV nucleocapsid (N), S, and membrane (M) proteins compared to healthy control sera (Fig. 1). SARS-CoV-2 N protein was unavailable at the time of publication and was not included. The receptor binding domain (RBD) of the SARS-CoV-2 S protein had overall weaker antibody binding signals but, nevertheless, was significantly more reactive with COVID-19 patient serum IgG and IgA than with control serum IgG and IgA. The signals shown in Fig. 1 are the base 2 logarithm of the raw intensities without normalization, since the background reactivity of each purified protein is different. The SARS-CoV-2 S and RBD as well as the SARS-CoV S, N, and M purified proteins had the largest mean differences between IgG binding of the negative and positive groups, and the differences are the most statistically significant (*t* test *P* values of <10^−5^). The same five antigens had the largest significant mean differences between IgA binding of the negative and positive groups. Only SARS-CoV-2 S and SARS-CoV N, however, had significant differential IgM binding between the COVID-19 patients and the control group. These results are in agreement with the enzyme-linked immunosorbent assays (ELISA) shown in Tables 2 and 3.

### SARS-CoV-2 protein fragments identify antigenic regions.

**FIG 2:**
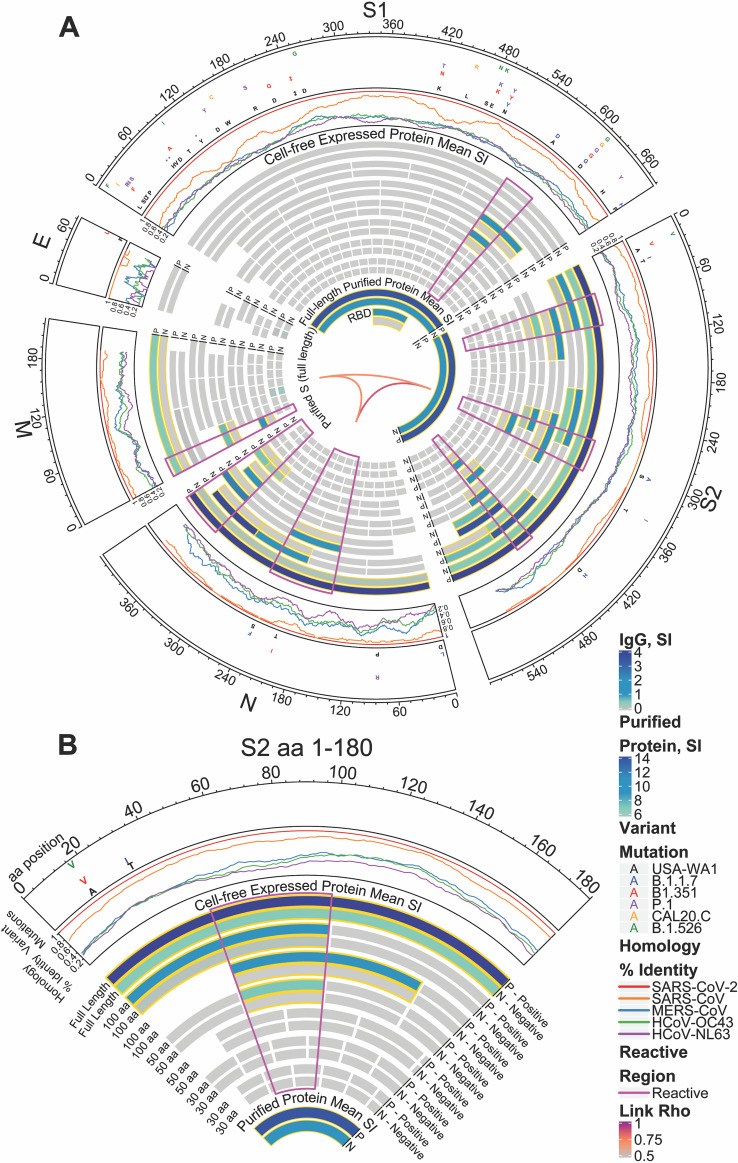
Reactivity of COVID-19 patient and healthy donor IgG to SARS-CoV-2 proteins and protein fragments. (A) The circular graphic maps the amino acid (aa) position of SARS-CoV-2 fragments, showing a heat map of IgG levels in each group for overlapping regions of different amino acid length. Proteins are indicated outside the circle plot, followed by a line graph showing the sequence homology of other CoVs with SARS-CoV-2 for each gene. Mutations, relative to the USA-WA1 strain, found in circulating mutant virus strains are shown in the next circular segment. Proteins and protein fragments produced *in vitro* are indicated by bars and show the length and position of each fragment in each protein. Each fragment is drawn twice and shows the group mean normalized log_2_ signal intensity of IgG binding to each fragment for COVID-19-positive samples (P) and negative-control sera (N). The purified full-length S protein and the receptor binding domain (RBD) are shown for comparison. IgG signal intensity is shown by color gradient, from gray to blue. Bar pairs shown with a gold outline represent significantly differential IgG binding between COVID-19 patients and healthy controls, defined as a mean log_2_ signal intensity of ≥0.1 in at least one group and a *t* test *P* value of ≤0.05. The regions of greatest reactivity for each protein are outlined in magenta. The Pearson’s correlation coefficients (“Rho”) between each full-length protein for IgG binding are shown as links between protein sectors in the center of the circle. (B) A slice of the circular graphic is amplified and labeled in more detail as a guide to interpreting the full figure. The first 180-aa sequence of S2 is shown.

Nine SARS-CoV-2 full-length proteins were produced by coupled *in vitro* transcription and translation (IVTT)—S, envelope (E), M, N, and open reading frames (ORFs) 3a, 6, 7a, 8, and 10. We used the same technique to produce overlapping 100-amino acid (aa), 50-aa, and 30-aa fragments of each of these 9 SARS-CoV-2 proteins and to produce the structural proteins and some accessory proteins of HCoV-NL63, HCoV-OC43, SARS-CoV, and MERS-CoV. Using amino acid start and end positions of each fragment within the protein, differential IgG reactivity between the COVID-19 and healthy donor groups was mapped in a circular heatmap for the structural proteins (Fig. 2). This analysis allowed us to identify antigenic regions in each SARS-CoV-2 structural protein.

The SARS-CoV-2 N protein showed the strongest reactivity in its carboxy-terminal 100-aa fragment, as well as in 50-aa fragments covering the same region. This region was recognized by IgG, IgA, and IgM with significant differential reactivity between COVID-19-positive patients and the healthy negative-control group (Fig. 2; see Fig. S1 in the supplemental material). The middle of the N protein also had a region recognized by IgG and IgA identified by two 100-aa fragments. This central region of the N protein is the location of two mutations found in circulating strains of SARS-CoV-2, S235F found in the UK strain B.1.1.7 and T205I found in the South African strain B.1.351. Together these two antibody-reactive regions encompass about two-thirds of the N protein that likely contains at least two epitopes.

The S1 protein also showed greatest IgG binding near its carboxy terminus, in the penultimate 100-aa fragment, aa 501 to 600 (Fig. 2). This antigenic region of S1 was defined further by IgG and IgA reactivity with the 50-aa fragment from aa 551 to 600. This region is also the site of a mutation found in strain B.1.1.7, A570D, and another mutation that was fixed early in the pandemic and is present in nearly all circulating strains of SARS-CoV-2, D614G. The region containing the RBD was not strongly reactive when produced by IVTT, but significant IgG, IgA, and IgM reactivity was detected to the purified RBD fragment (Fig. 2 and Fig. S1). Moreover, the RBD is the site of mutations in several circulating strains of SARS-Cov-2—K417N in B.1.351 and K417T in the Brazilian strain P.1, as well as L452R in the California strain CAL20.C. The S2 protein of SARS-CoV-2 was highly antigenic with three regions of strong IgG, IgA, and IgM binding and differential reactivity with full-length, 100-aa and 50-aa fragments. Only the region near the carboxy terminus, however, was also reactive as a 30-aa fragment. This reactive 30-aa fragment, from aa 451 to 480 of S2 (1,136 to 1,165 of S), therefore likely defines a linear IgG epitope in this highly antigenic protein. Notably, an epitope in the central S2 antigenic region was differentially reactive for IgG and IgA but showed equal levels of IgM reactivity in 100-aa and 50-aa fragments, perhaps indicating a region of cross-reactivity for IgM produced by memory B cells reactive with an endemic human coronavirus. This central antigenic region of the S2 protein includes the site of a mutation in the B.1.1.7 strain, S297A (S982A of S).

An additional short epitope was found in the amino terminal 30-aa fragment of the SARS-CoV-2 M protein. This short fragment was highly reactive with COVID-19 patient serum IgG compared to healthy donor serum IgG, while larger fragments containing it, and the full-length M protein, were not as highly discriminatory for COVID-19 patient sera. The SARS-CoV-2 E protein had only one 30-aa fragment that showed low-level reactivity with IgA and IgM (Fig. S1), in both the COVID-19-positive and -negative groups.

The antigenic regions of SARS-CoV-2 structural proteins we identified did not correlate with homology between SARS-CoV-2 and other human coronaviruses (percentage amino acid sequence identity shown in outer track of Fig. 2). There was a moderate to high level of correlation between antibody reactivity with S2, N, and M proteins produced *in vitro*, particularly for IgG (Pearson’s correlation coefficient shown in the inner links of Fig. 2). Less reactivity was seen in nonstructural proteins, but significant reactivity of COVID-19 patient IgG and IgA compared to that of negative-control IgG and IgA was e identified in fragments of the 3a and 7a accessory proteins (Fig. S2).

### Individual antibody response profiles to antigenic regions of SARS-CoV-2 and other human coronaviruses.

**FIG 3:**
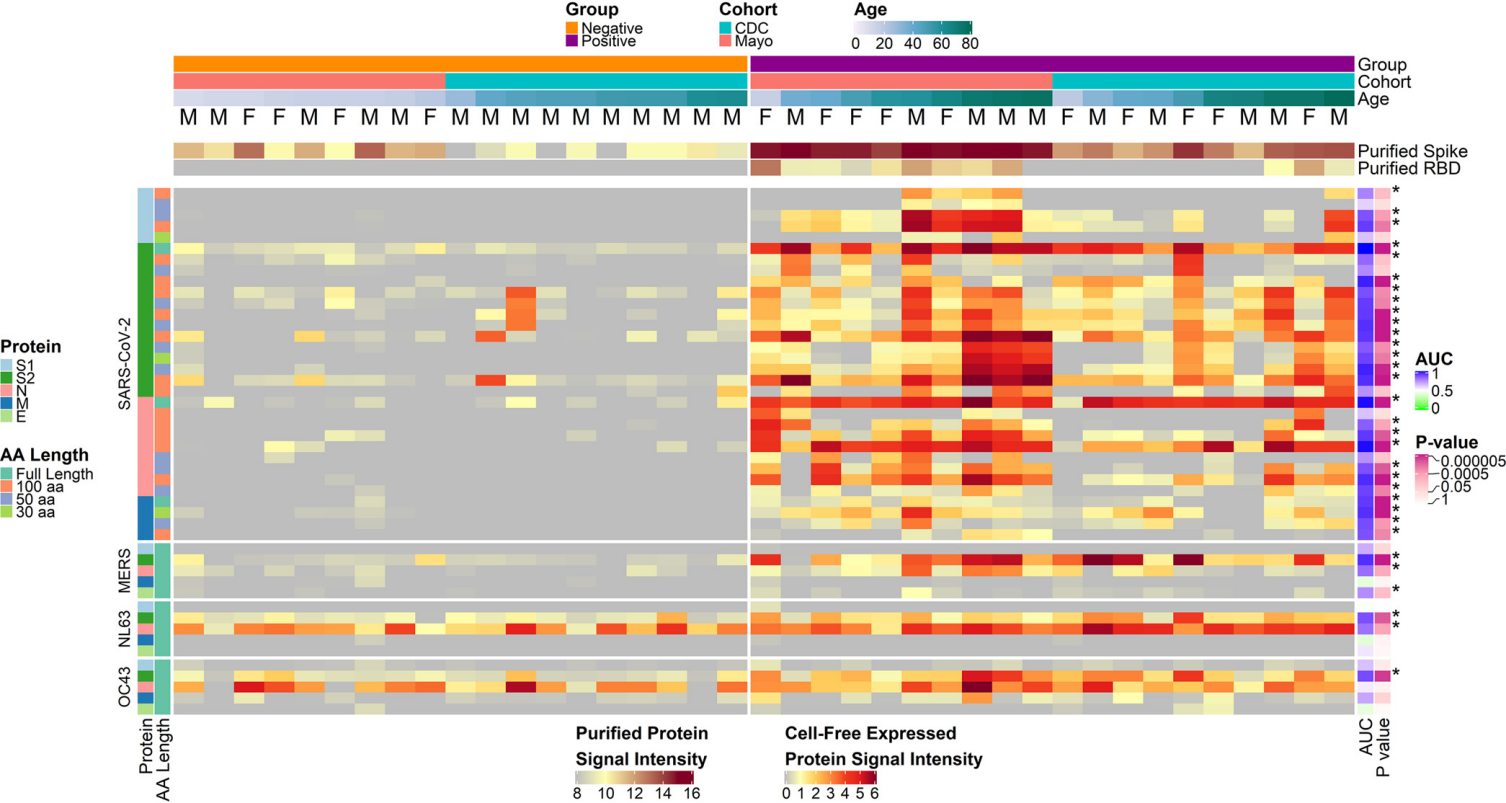
COVID-19-positive and -negative sample IgG reactivity to coronavirus proteins and protein fragments produced *in vitro*. The heatmaps present the signals of IgG binding to individual proteins and protein fragments within the antigenic regions of SARS-CoV-2, as well as the full-length structural proteins of MERS-CoV, HCoV-NL63, and HCoV-OC43 for individual samples. Columns represent serum samples ordered by increasing age within group and cohort, and rows represent proteins or protein fragments, including 32 SARS-CoV-2 proteins or fragments and 5 proteins each of MERS-CoV, HCoV-OC43, and HCoV-NL63. IgG signal intensity is shown on a color scale from gray to red. Sample information is overlaid above the heatmaps and includes sex (M/F), group (negative or positive), cohort (CDC or Mayo), and age (years). Protein/fragment information is annotated to the left of the heatmaps and includes the virus, the full-length protein name, and the amino acid length of the protein fragments (“AA Length,” as full length, 100, 50, or 30 aa). The receiver operating characteristic area under the curve (AUC) and the unadjusted *t* test *P* value for each protein between negatives and positives are shown to the right of the heatmap. Asterisks next to the *P* values represent adjusted *P* values of <0.05.

Individual IgG responses to the antigenic regions of SARS-CoV-2 proteins identified by reactivity with protein fragments varied substantially between individuals, as they did for the structural proteins of other human coronaviruses (Fig. 3). Individual variation is also evident in IgG responses against all the SARS-CoV-2 S1, S2, N, and M protein fragments on the array (Fig. S3). Within the antigenic regions, some fragments, particularly 30-aa fragments, were nonreactive with COVID-19 patient sera, but others were reactive in a subset of individuals. Heterogeneity was higher and overall signal intensities were lower for IgA and IgM than for IgG (Fig. S4). There were no significant associations between age and sex with antibody levels in the positive group after adjustment for the false-discovery rate for any of the three isotypes (Table S1).

IgG from most negative controls and all COVID-19 patients was significantly reactive (normalized log_2_ signal intensity, ≥1.0) with the HCoV-NL63 N protein; 17 of 19 negative sera and all 20 patient sera were reactive (proportion test *P* value of 0.4; Fig. 3). Similarly, most negative-control serum IgG and all patient serum IgG samples were significantly reactive with HCoV-OC43 N protein—15 of 19 negative sera and all 20 patient sera (*P = *0.1). In contrast, IgG from only two control subjects reacted with the SARS-CoV-2 full-length N protein, while nearly all of the patients’ serum IgG reacted—19 of 20 reactive (*P = *6.8 × 10^−7^). Moreover, reactivity of the negative-control serum IgG with fragments of the SARS-CoV-2 N protein occurred rarely and exclusively in the C-terminal region of the protein (1 of 19 reactive), while COVID-19 patient serum IgG reacted frequently with fragments in the central region (12 of 20; *P = *2.1 × 10^−4^) as well as the C-terminal region of the protein (19 of 20; *P = *1.3 × 10^−7^).

HCoV S2 proteins were reactive with COVID-19 patient IgG at a much higher frequency than in the controls for both HCoV-NL63 (16 of 19 and 5 of 20 positives, respectively; *P = *2.4 × 10^−3^) and HCoV-OC43 (18 of 19 and 4 of 20, respectively; *P = *5.9 × 10^−5^). The higher frequencies in the COVID-19-positive subjects provide strong evidence of increased responses due to their exposure to SARS-CoV-2. Some negative-control subject’s IgG reacted with the C-terminal (4 of 19) or central regions (4 of 19) of the SARS-CoV-2 S2 protein, but none reacted with the N-terminal region; this includes one individual who had unique reactivity to the 100-aa SARS-CoV-2 S2 fragment, 401 to 500 (S 986 to 1085; Fig. S3). By ELISA, this serum had a signal to threshold (S/T) ratio of 0.94, which was just below the positivity threshold of 1.0 and much higher than that of other healthy donor sera. This reactivity was unique among negative-control donors but did not directly translate to reactivity with OC43 or NL63 full-length S proteins. Overall, COVID-19 patient serum IgG reacted with the SARS-CoV-2 S2 protein C-terminal (19 of 20; *P = *1.3 × 10^−5^), central (17 of 20; *P = *2.3 × 10^−4^), and/or N-terminal (12 of 20; *P = *2.1 × 10^−4^) 100-aa fragments much more frequently than prepandemic negative-control sera.

The reactivity of COVID-19 patient serum IgA compared to IgA of healthy donor sera was similar to results obtained for IgG. The IgA results had lower statistical significance than the IgG results, however, likely due to the lower concentration of IgA in serum compared to IgG. Nevertheless, many of the same proteins were the most differentially reactive with COVID-19 patient serum IgA compared to healthy donor serum IgA, including the N and S proteins and RBD of SARS-CoV-2 as well as the N, S, and M proteins of SARS-CoV with *t* test *P* values ranging from 2.1 × 10^−6^ to 1.1 × 10^−3^ (Fig. S4). The COVID-19 patient sera used in this study had less coronavirus reactive IgM than IgG or IgA, perhaps because the samples were obtained during the convalescent phase of disease. Nevertheless, significantly greater IgM reactivity was seen in patient sera compared to control donor sera for four proteins and two protein fragments produced *in vitro* (Fig. S4). These were the N, S2, and M proteins of SARS-CoV-2, the MERS-CoV N protein, the carboxy-terminal 100-aa fragment of the SARS-CoV-2 N protein, and the amino terminal 30-aa fragment of the SARS-CoV-2 M protein.

A library of 587 peptides, 15 to 20 aa in length, from the epidemic SARS-CoV (covering S, N, M, and E proteins) and 2 endemic human coronaviruses (covering S protein) was printed on the multicoronavirus microarray at the same concentration as full-length purified recombinant proteins. The peptides, however, showed lower antibody reactivity than full-length proteins or protein fragments of 30, 50, or 100 aa (data not shown). Exceptionally, a single 17-aa peptide from HCoV-OC43 S protein with sequence CSKASSRSAIEDLLFDK spanning residues 905 to 921 had approximately 3.5-fold higher mean reactivity with COVID-19 patient sera (*P = *0.001, not significant after adjustment for the false-discovery rate). This peptide mapped to the SARS-CoV-2 sequence PSKPSKRSFIEDLLFNK at residues 124 to 140 of S2 (809 to 825 of S protein) with identical residues in 12 of 17 positions.

**FIG 4:**
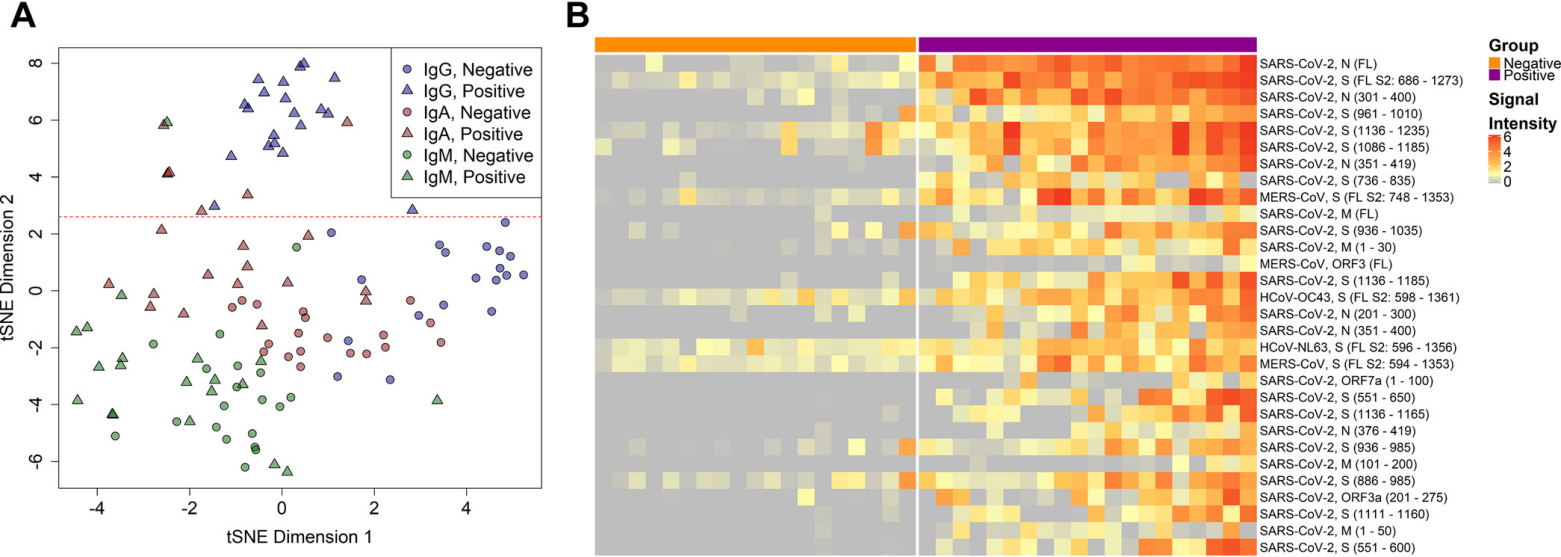
IgG responses give the best delineation of COVID-19 patient sera from healthy donor sera. (A) The IgG, IgA, and IgM responses against the 30 most reactive IVTT proteins by means of all samples and isotypes were projected for each sample across two dimensions using t-distributed stochastic neighbor embedding (tSNE). Points represent individual samples and are colored according to the isotype measurement. The shape represents the group in which the sample belonged, either the negative healthy donor group or the positive COVID-19 patient group. The horizontal red dashed line (*y* = 2.6) separates the IgG responses in negative and positive individuals. (B) The heatmap shows the 30 most differentially reactive IgG responses to IVTT proteins between the negative and positive groups. Columns represent serum samples, separated by group with colored headers. Rows represent full-length or fragmented proteins produced by cell-free expression *in vitro*. The protein annotations to the right of the heatmap denote the virus, protein, and in parentheses, the amino acid range of the fragment or full-length “FL” protein. Normalized log_2_ signal intensity is displayed on a gray to red color scale.

To visualize the relative importance of antibody isotype binding in differentiating COVID-19-positive sera from negative sera, the samples were projected in two dimensions for each isotype using t-distributed stochastic neighbor embedding (tSNE; Fig. 4A), a nonlinear machine learning dimensionality reduction method which clusters together similar sets of multidimensional data. The 30 most reactive proteins for all isotypes were selected for this analysis to reduce the effect of differing isotype background levels that would be notable in low-reactivity proteins (Fig. 4B). Each of the isotypes clusters separately, but only IgG gave a clear delineation of positives and negatives (at ∼2.6 in tSNE dimension 2).

The full-length SARS-CoV-2 N and S2 proteins as well as several fragments of both proteins had the top nine largest mean differences in IgG reactivity between COVID-19 patients and healthy controls (Fig. 4B). These results were also statistically significant, with *t* test *P* values ranging from 2.1 × 10^−6^ to 4.3 × 10^−2^ (Table S1). Antibody responses to HCoV-NL63, HCoV-OC43, and MERS-CoV proteins were also among the 30 most discriminatory antigens for differentiating COVID-19 patients from control donors due to high reactivity with COVID-19-positive sera, while also demonstrating a considerable reactivity with negative sera. Nearly all the same epitopes and regions of reactivity found for IgG were recapitulated by IgA reactivity when reactivity to the overlapping 100-aa, 50-aa, and 30-aa protein fragments was analyzed (Fig. S4). This includes the epitopes mapped in the SARS-CoV-2 N, S1, S2, and M proteins (Fig. 2).

### Correlation of SARS-CoV-2 and endemic human coronavirus responses.

**FIG 5:**
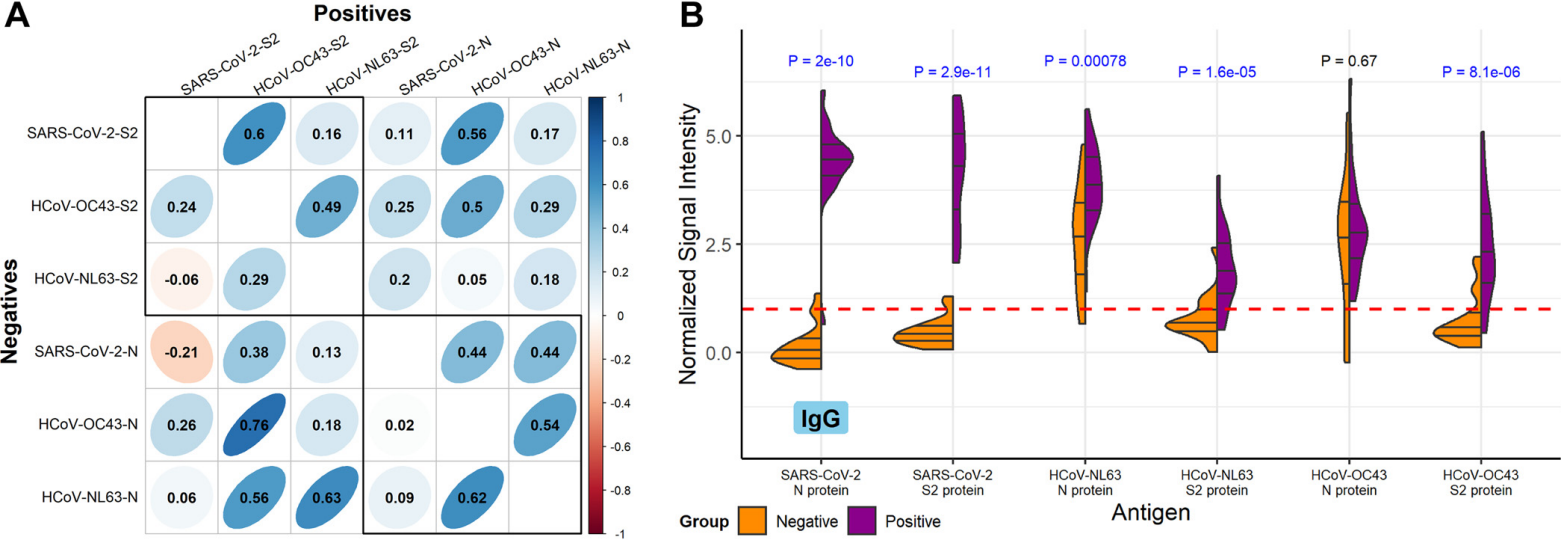
Correlation and concordance between IgG responses to SARS-CoV-2 and endemic human coronavirus N and S2 proteins. (A) The correlogram shows the Pearson’s correlation coefficient (*ρ*) between IgG normalized signal intensity to SARS-CoV-2, HCoV-OC43, and HCoV-NL63 N and S2 full-length proteins produced *in vitro*. The lower half of the diagonal shows the correlation between reactivity of sera in the negative group, and the upper half of the diagonal shows the positive group serum correlations. The color scale indicates positive correlation in darker shades of blue and negative correlation in darker shades of red, and *ρ* is overlaid on each comparison. Additionally, the narrowness and slope of the ellipses represent increasing positive or negative correlation. Boxes are drawn around the intra-S2 and intra-N protein comparisons. (B) The split violin plot shows the normalized log_2_ IgG signal intensity distribution for each N and S2 protein produced *in vitro*. Within each half-violin are three lines representing the interquartile range and the median. Above each split violin is the Wilcoxon rank sum *P* value, colored blue for significant *P* values below 0.05. The red dashed line represents the 1.0 seropositivity cutoff.

By comparing the correlation between antibody responses to the S2 and N proteins of SARS-CoV-2 with responses to the S2 and N proteins of endemic human coronaviruses, in both COVID-19-positive and -negative sera, we can estimate to what extent antibody responses to SARS-CoV-2 are the result of *de novo* immune responses or of boosting preexisting immunity. There were significantly stronger correlations between SARS-CoV-2 S2 protein IgG and HCoV-OC43 S2 proteins in the positive group (Pearson’s correlation coefficient, *ρ* = 0.6) than the negative group (*ρ* = 0.24; Fig. 5A, top left). In the negative group, SARS-CoV-2 N protein IgG had no correlation with HCoV-OC43 N protein (*ρ* = 0.02) or HCoV-NL63 N protein (*ρ* = 0.09), whereas the correlations in the positive group were higher; HCoV-OC43 and HCoV-NL63 had a *ρ* value of 0.44 with SARS-CoV-2 N protein. These results suggest that *de novo* responses to SARS-CoV-2 S2 and N proteins are predominant. HCoV-OC43 and HCoV-NL63 N protein reactivity exhibited strong correlations in both positive and negative groups; *ρ* = 0.54 and *ρ* = 0.62, respectively. S2 protein reactivity correlations between these endemic human coronaviruses, however, were lower in the negative group than the positive group; *ρ* = 0.29 and *ρ* = 0.49, respectively. Further inspection of the IgG correlation scatterplot matrix (Fig. S5) showed an outlier sample in the CDC positive group for SARS-CoV-2 N protein, which had a normalized signal intensity of 0.65, 3 normalized signal intensity units lower than the next lowest sample. This had an outsized effect on SARS-CoV-2 N protein correlations; for example, removal of the sample increased correlation between SARS-CoV-2 N protein and HCoV-OC43 N protein among COVID-19-positive IgG, from 0.44 to 0.75 (data not shown). Differential IgG reactivity between the COVID-19-positive and -negative groups was also observed with the S2 and N proteins of SARS-CoV-2, HCoV-OC43, and HCoV-NL63. Positive COVID-19 patient sera had significantly higher IgG levels to S2 and N than the negative healthy donor sera for all three coronaviruses (Fig. 5B), with the exception of HCoV-OC43 N protein; this protein also showed higher IgA reactivity in the negatives (Fig. S6).

### Correlation of multi-coronavirus protein microarray responses with ELISA and virus neutralization assays.

**FIG 6:**
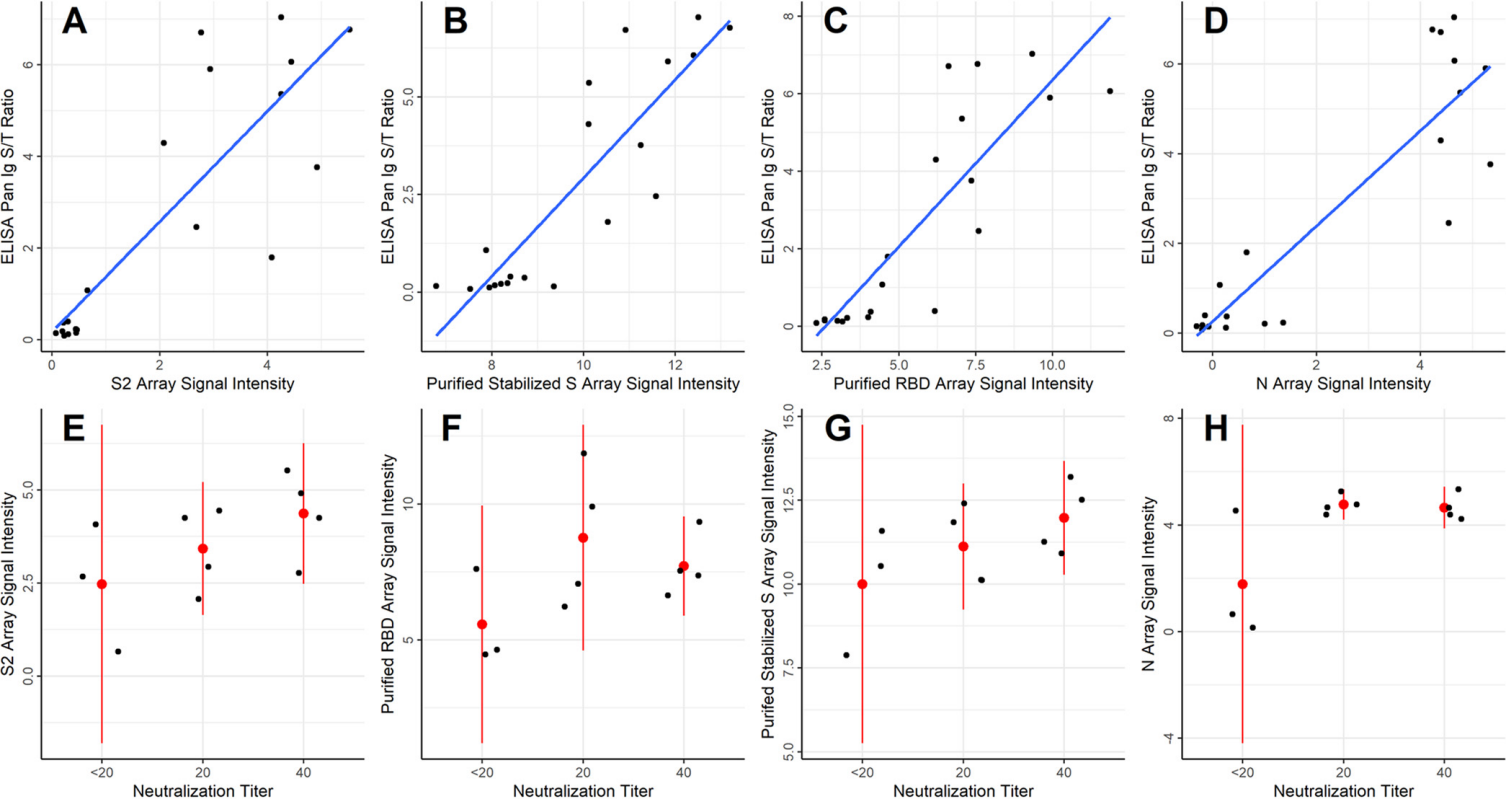
Correlation of IVTT and purified protein microarray results with ELISA and virus neutralization assays. (A to D) The scatterplots show the SARS-CoV-2 S protein-based ELISA Pan Ig signal/threshold ratio (*y* axis) plotted against the protein microarray log_2_ normalized IgG signal intensity for S2 and N proteins produced *in vitro*, as well as for the stabilized, purified full-length S protein and the purified RBD fragment of S1 protein (*x* axis), respectively. The blue lines were fit to the data using linear regression. (E to H) The dot plots show individual values of each patient for the protein microarray log_2_ normalized IgG binding intensity (*y* axis) of the four proteins shown in panels A to D at each neutralization titer (*x* axis). Red dots are plotted at the means of each stratum, and the red lines represent the 95% confidence intervals.

S protein-based ELISA results from the CDC cohort, taken on all COVID-19 and healthy negative donor samples, were compared with IgG reactivity in the protein microarray data by Pearson’s correlation coefficient for the highly reactive IVTT S2 protein (*ρ* = 0.85), IVTT N protein (*ρ* = 0.9), purified recombinant full-length S protein (*ρ* = 0.88), and purified recombinant RBD (*ρ* = 0.85), shown in Fig. 6A to D. The data clustered separately for negative responders and positive responders for all proteins. Virus neutralization titers were only available for the CDC COVID-19 patients and one healthy donor sample that tested near the 1.0 cutoff for ELISA reactivity (*n* = 11). In all cases, neutralization activity was low, with positive neutralization titers at dilution factors of 20 or 40. Despite the low values and few samples, a trend was observed using linear regression for IVTT S2 (β = 6.5, *P = *0.076), IVTT N (β = 6, *P = *0.036), and stabilized purified S (β = 6.3, *P = *0.077) (Fig. 6E to H). There was no association, however, of neutralization activity with IgG reactivity to purified RBD (β = 2.8, *P = *0.27). The linear regression models were specified with values of 0 for titers of <20. However, since the true titer is between 0 and 20, neutralization was also modeled as an ordinal variable using ordinal logistic regression. Similar results were obtained for IVTT S2 and stabilized purified S, whereas association with IVTT N protein was no longer significant. The complete correlation results for all proteins are shown in Table S1.

## DISCUSSION

In this study of 20 COVID-19 patients, the strongest antibody responses to the SARS-CoV-2 proteins used on this array, for all antibody isotypes, were directed to the N and S2 proteins as has been previously seen in other studies (4, 7, 20, 21). We also detected antibody responses to S1, M, and accessory proteins 3a and 7a. Moreover, we localized regions of each of these SARS-CoV-2 proteins to which antibodies bound, by antibody reactivity with overlapping protein fragments of three different lengths—100, 50, and 30 aa. Our results were internally consistent in that reactive proteins had more reactive fragments than nonreactive proteins and 100-aa reactive fragments contained reactive 50-aa fragments and sometimes they also contained reactive 30-aa fragments. We found little reactivity of COVID-19 patient sera with 13- to 20-aa peptides from SARS-CoV S, M, E, or N, HCoV-OC43 S, or HCoV-NL63 S with the exception of one S2 peptide from HCoV-OC43.

Many previous publications have predicted B cell epitopes in SARS-CoV-2 proteins using a variety of immunoinformatic approaches (22–25). Crooke et al. (22) predicted 26 potential linear B cell epitopes in the S protein, 14 potential epitopes in the N protein, and 3 potential epitopes in the M protein. We noted antibody reactivity with regions containing some, but not all, of these predicted epitopes. In particular, of the top six predicted B cell epitopes in the S protein we found significantly stronger reactivity with COVID-19 patient sera than with healthy donor sera for regions containing three epitopes—DIADTT (residues 568 to 573 near the carboxy terminus of S1), PPIKD (residues 792 to 796 near the amino terminus of S2), and VYDPLQPELDSF (residues 1137 to 1148 near the carboxy terminus of S2). The other three top predicted B cell epitopes of the S protein, residues 405 to 428, 440 to 450, and 496 to 507, were not in highly reactive regions of the S protein in our experiments, perhaps due to the overall low reactivity of the S1 protein except for its carboxy-terminal region or a need for native structure not found in protein fragments produced *in vitro*. Similarly, we found COVID-19-specific reactivity for regions including 9 of the 14 B cell epitopes in the N protein and 1 of 3 B cell epitopes in the M protein predicted by Crooke et al.

A few other groups have used protein or peptide arrays to map antibody reactivity to SARS-CoV-2 protein (21, 26–29). Two studies included full-length purified structural proteins from SARS-CoV-2, other human coronaviruses, and diverse human retroviruses (21, 26). Their results are consistent with ours but do not include accessory proteins or the ability to map reactive regions in each protein. Several groups used peptides to map epitopes in the SARS-CoV-2 S protein (27–29); Li et al. (27) found 4 epitopes defined by 12 amino acid peptides, 3 of which are in regions of antibody reactivity that we found. Poh et al. (28) found 2 epitopes defined by 18 amino acid peptides. Both are in regions of antibody reactivity that we described here. Finally, Zhang et al. (29) used 15 amino acid peptides overlapping by 5 aa covering the whole SARS-CoV-2 proteome, plus full-length N and E as well as 5 truncated forms of S to map IgM and IgG responses of acute COVID-19 patients (median 4 days post-onset of symptoms). They found more robust IgM responses than we did, since their specimens were collected earlier during infection. Zhang et al. identified five peptides as the most specific for COVID-19 patient IgG binding compared to controls, two in the S protein, two in N, and 1 in ORF-1ab. Both S protein peptides are in regions where we found IgG reactivity; one is in the N-terminal region of reactivity we found in S2, and the other is in the central reactivity region of S2. The N peptides of this group were not in a reactive region in our work, and we did not assay antibody reactivity of the ORF-1ab polyproteins.

Two groups published epitope maps of SARS-CoV-2 using phage display (30, 31). One group analyzed 56 aa and 20 aa fragments of the SARS-CoV-2 proteome, while the other group analyzed 38 aa fragments of the proteome. Both studies also included other human coronaviruses and used COVID-19 patient sera and control sera to identify specifically reactive epitopes in the SARS-CoV-2 proteome. Their data are largely in agreement with data presented here. Both studies found the greatest reactivity of COVID-19 patient sera in the S2 and N proteins. Moreover, the epitopes they mapped overlapped with the ones we found here by different methods.

SARS-CoV-2-infected subjects have much higher antibody levels to SARS-CoV-2 proteins, primarily N and S, than uninfected individuals, but it is also clear that even in the small sample sets evaluated here, some SARS-CoV-2-naive individuals have substantial preexisting antibody to some epitopes of these two proteins. These preexisting antibody levels have been shown to vary according to many different factors, including age, and may therefore have relevance to the clinical course of disease (32–34). Antibody reactivity of both positive and negative sera with endemic HCoVs and MERS-CoV was strongest for the S2 and N proteins as expected due to their abundance and conservation. Correlations between antibody responses to SARS-CoV-2 S2 and N proteins and to HCoV S2 and N were much stronger in COVID-19 convalescent-phase sera compared to negative sera, indicating that new cross-reactive responses to SARS-CoV-2 predominated over preexisting antibodies to HCoVs that cross-reacted with SARS-CoV-2 proteins.

The multi-coronavirus protein array is a tool that can help us improve our understanding of the immune response to SARS-CoV-2 and other coronaviruses. With these first two sets of convalescent-phase sera provided by the Mayo Clinic and the CDC, we have shown that SARS-CoV-2-naive subjects have clearly measurable cross-reactive antibody to the whole N and S2 proteins and that this reactivity is limited to specific epitopes. Importantly, there are epitopes that are more specific to SARS-CoV-2, that might serve as useful biomarkers of infection. Conversely, we have shown that infection with SARS-CoV-2 elicits or boosts the level of antibodies that bind to the N and S2 proteins of other coronaviruses, including SARS-CoV, MERS-CoV, HCoV-NL63, and HCoV-OC43.

The limitations of our study are the small sample size and the inclusion of only convalescent-phase samples. Despite these limitations, we identified clear differences in the antibody response from COVID-19 patients and healthy, nonexposed controls. The ideal data set to further investigate associations between preexisting antibody to specific epitopes and protection from severe disease would be longitudinal, with at least a preexposure sample, an acute-phase sample, and a convalescent-phase sample from each subject. Inclusion of samples from COVID-19 patients with a range of clinical symptoms will also provide an important comparison. In upcoming projects, we are seeking to analyze these types of samples paired with detailed clinical data on disease outcomes ranging from asymptomatic to fatal to further improve our understanding of the complex role antibodies play in SARS-CoV-2 infection. It may also prove interesting to test convalescent plasma samples, especially given the variable efficacy results that have been reported in the literature (16–19). An assay providing more granular detail on the humoral response in these samples, such as the protein microarray described here, may provide valuable insights into factors that determine the effects of convalescent plasma treatment.

## MATERIALS AND METHODS

### Protein microarray analysis of serum samples.

The first-generation multi-coronavirus protein microarray, produced by Antigen Discovery, Inc. (ADI, Irvine, CA, USA), included 935 full-length coronavirus proteins, overlapping 100-, 50-, and 30-aa protein fragments, and overlapping 13- to 20-aa peptides from SARS-CoV-2 (WA-1), SARS-CoV, MERS-CoV, HCoV-NL63, and HCoV-OC43. Purified proteins and peptides were obtained from BEI Resources. All these coronavirus proteins were produced in Escherichia coli except the SARS-CoV-2 and SARS-CoV S proteins, which were made in Sf9 insect cells, and the SARS-CoV-2 RBD, made in HEK-293 cells. Other proteins and protein fragments were expressed using an E. coli *in vitro* transcription and translation (IVTT) system (rapid translation system; Biotechrabbit, Berlin, Germany) and printed onto nitrocellulose-coated glass AVID slides (Grace Bio-Labs, Inc., Bend, OR, USA) using an Omni Grid Accent robotic microarray printer (Digilabs, Inc., Marlborough, MA, USA). Microarrays were probed with sera and antibody binding detected by incubation with fluorochrome-conjugated goat anti-human IgG, IgA, or IgM (Jackson ImmunoResearch, West Grove, PA, USA, or Bethyl Laboratories, Inc., Montgomery, TX, USA). Slides were scanned on a GenePix 4300A high-resolution microarray scanner (Molecular Devices, Sunnyvale, CA, USA), and raw spot and local background fluorescence intensities, spot annotations, and sample phenotypes were imported and merged in R (35), in which all subsequent procedures were performed. Foreground spot intensities were adjusted by subtraction of local background, and negative values were converted to 1. All foreground values were transformed using the base 2 logarithm. The data set was normalized to remove systematic effects by subtracting the median signal intensity of the IVTT controls for each sample. With the normalized data, a value of 0.0 means that the intensity is no different than the background, and a value of 1.0 indicates a doubling with respect to background. For full-length purified recombinant proteins and peptide libraries, the raw signal intensity data were transformed using the base 2 logarithm for analysis.

### Control sera and COVID-19 patient samples.

COVID-19-positive and pre-COVID-19 negative-control sera provided by the CDC were acquired from commercial laboratories or through partnership with Emory University. Samples were provided with only clinical and demographic information retained. The majority of samples (7/10) were from patients that were not hospitalized, with blood collected between 26 and 60 days post-symptom onset. Negative-control sera were collected pre-COVID-19, in the fall of 2019. This activity was reviewed by the CDC and was conducted consistently with applicable federal law and CDC policy (45 C.F.R. part 46, 21 C.F.R. part 56). The COVID-19-positive samples provided by the Mayo Clinic were deidentified residual sera from clinical testing with only age and sex information available. The COVID-19-negative samples were collected pre-COVID-19 pandemic, between 2005 and 2012. These samples were from participants in prior Mayo Clinic vaccine studies who had provided informed consent for future use of their biospecimens. The original blood collection was collected through Mayo Clinic IRB-approved protocols. Samples were tested for SARS-CoV-2-specific antibodies and the presence of neutralizing antibodies as described below.

### Enzyme linked immunosorbent assay (ELISA).

The CDC provided samples were tested using an enzyme-linked immunosorbent assay (ELISA) against the prefusion stabilized ectodomain of SARS-CoV-2 spike protein (36). This validated assay has been shown to have sensitivity and specificity of 96% and 99%, respectively (37). Briefly, plates were coated with purified spike protein and incubated overnight at 4°C followed by 37°C incubation steps and subsequent phosphate-buffered saline + 0.05% Tween 20 (PBST) washings with 2.5× Stabilcoat blocker (Surmodics), 1:25 to 1:1,600 diluted serum in 1× PBST + 5% skim milk for 1 h, 1:2,000 goat anti-human Ab conjugated to horseradish peroxidase (KPL) for 1 h, and ABTS peroxidase substrate for 30 min. Reactions were then quenched with stop solution. Plates were read at 405 nm and 490 nm, with resulting optical densities (ODs) calculated as 490 nm to 405 nm absorbance for each sample and minus PBS-only-coated wells. Results are reported as a ratio of the calculated sample OD/cutoff threshold OD (signal/threshold, or S/T); values of >1.0 are defined as positive. The Mayo Clinic COVID-19-positive samples were tested using an IgG SARS-CoV-2 spike protein-specific ELISA (EuroImmune, Inc.) performed according to the manufacturer’s recommendations. This validated assay has been shown to have sensitivity and specificity of 90% and 100%, respectively (38). Results are reported as a ratio of the sample OD/calibrator OD (signal/calibrator, or S/C); values of >1.1 are defined as positive.

### Neutralization assay.

All SARS-CoV-2 microneutralization assays (MNT) were performed following biosafety level-3 precautions, using a SARS-CoV-2 clinical isolate. The WA1 strain of SARS-CoV-2 was employed using a modified version of a previously established protocol. A total of 27 Vero cell suspensions (ATCC CCL-81) were prepared at 2.2 × 10^5^ to 2.5 × 10^5^ cells/ml in Dulbecco modified Eagle medium (DMEM; Thermo Fisher; catalog no. 11965118) plus 10% fetal bovine serum (FBS, defined; HyClone; catalog no. SH30070.03) (heat-inactivated at 56°C for 30 min) plus 2× antibiotic-antimycotic (Thermo Fisher; catalog no. 15240062) plus 2× penicillin-streptomycin (Thermo Fisher; catalog no. 15140122) immediately before use. Sera were 2-fold serial diluted in serum-free DMEM in a 96-well flat-bottom plate, from 1:10 to 1:320, in triplicate, to a final volume of 50 μl/well. Then 50 μl SARS-CoV-2 was added to each well, such that final serum dilution titers ranged from 1:20 to 1:640. After a 30-min incubation at 37°C and 5% CO_2_, 100 μl of Vero cells in suspension were added to each well, for a final concentration of 2.2 to 2.5 × 104 cells/well. After 5 days cells were stained and fixed with crystal violet fixative (0.15% crystal violet, 2.5% ethanol, 11% formaldehyde, 50% PBS, 0.01 M pH 7.4). The endpoint concentration at which antibodies were determined to be neutralizing for SARS-CoV-2 infection was the lowest concentration of antibody at which 3 replicate wells were protected against virus infection.

### Statistical analysis.

Student’s *t* tests were used for comparison of the individual antibody response means between the positive and negative groups. Comparison of the medians was done using Wilcoxon’s rank sum test. The area under the receiver operating characteristics curve (AUC) was calculated to estimate delineation of groups for each antigen. The tSNE analyses were calculated after 25,000 iterations with a perplexity parameter of 30 using the R package Rtsne (39). Comparisons of the proportions of responders to each protein between groups was done using two-proportion *z* tests implemented by the prop.test function in R. Correlation between antibody features and between protein microarray and ELISA measurements used Pearson’s correlation coefficient (*ρ*), and association between antibody measurements and sample information such as sex, age, and cohort were modeled using linear regression. The association of specific antibody responses with virus neutralization titers was estimated using linear regression, with the values below detection levels (<20) coded as zero, or by converting neutralization titers to ordinal values and estimating the proportional odds ratio by ordinal logistic regression, whereby *P* values were estimated by comparing the *t* value against the standard normal distribution. Adjustment for the false-discovery rate was performed using the p.adjust function in R (40). Data visualization was performed using the circlize (41), ComplexHeatmap (42), ggplot2, heatmap2, and corrplot (38) packages in R. Unadjusted *P* values were shown in graphics.
