# Supplementary material for: Mapping SARS-CoV-2 Antibody Epitopes in COVID-19 Patients with a Multi-Coronavirus Protein Microarray
Source: Microbiol Spectr. 2021 Oct 27;9(2):e01416-21. doi: 10.1128/Spectrum.01416-21 (PMC8549749; doi:10.1128/Spectrum.01416-21)
Supplement: SUPPLEMENTAL FILE 1 — Supplemental material. Download SPECTRUM01416-21_Supp_1_seq10.pdf, PDF file, 2.1 MB [file spectrum01416-21_supp_1_seq10.pdf]

Mapping SARS-CoV-2 Antibody Epitopes in COVID-19 Patients with a Multi-Coronavirus Protein Microarray

Supplemental Figures

Figure S1

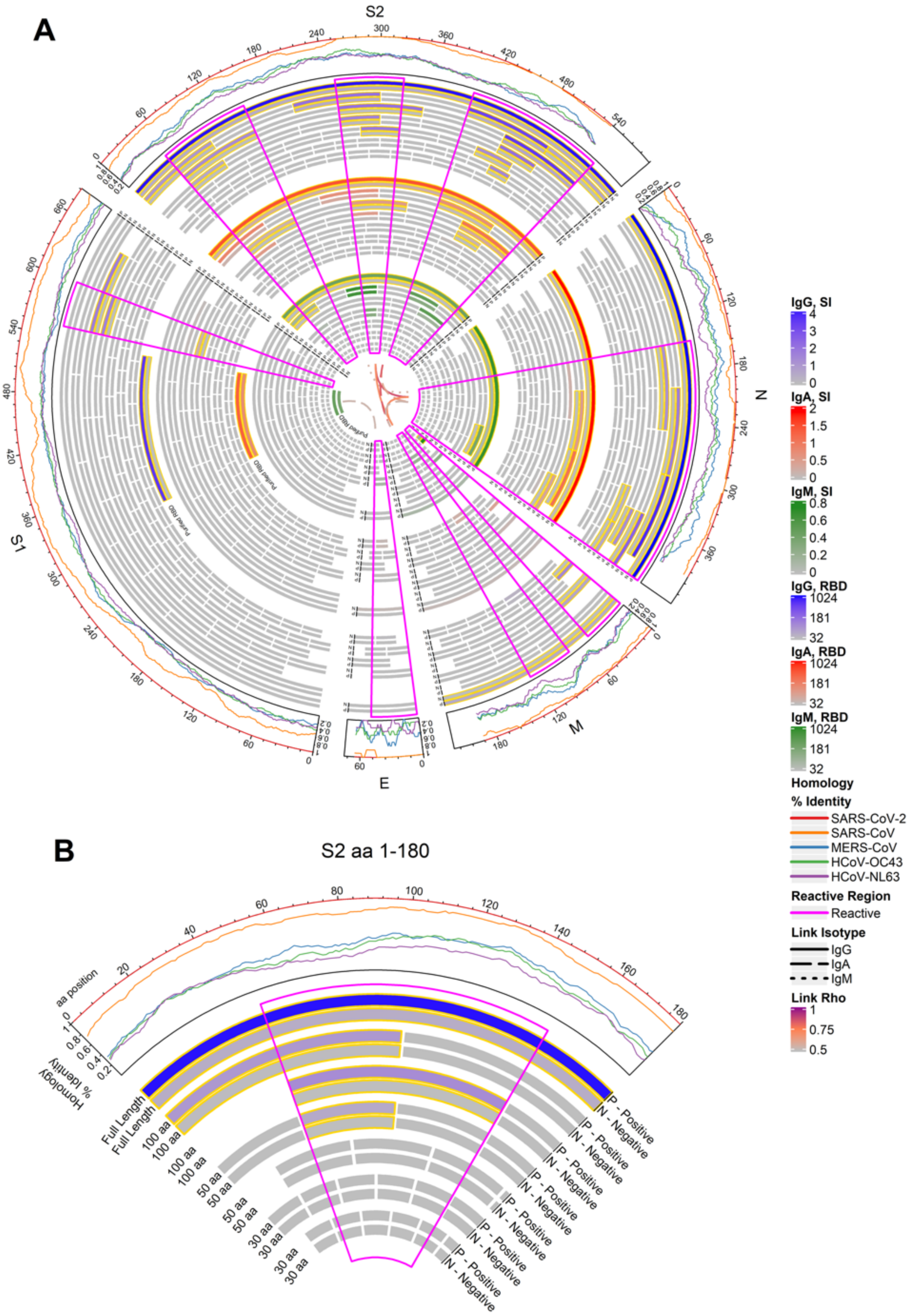
A
S2
S1
N
M
E
IgG, SI
IgA, SI
IgM, SI
IgG, RBD
IgA, RBD
IgM, RBD
Homology
% Identity
SARS-CoV-2
SARS-CoV
MERS-CoV
HCoV-OC43
HCoV-NL63
Reactive Region
Reactive
Link Isotype
IgG
IgA
IgM
Link Rho
B
S2 aa 1-180
aa position
Homology % Identity
Full Length
100 aa
50 aa
30 aa
P - Positive
N - Negative

Figure S1. Reactivity of COVID-19 patient and healthy donor IgG (outer band of bars), IgA (middle band) and IgM (inner band) to SARS-CoV-2 proteins and protein fragments. (A) The circular graphic maps the amino acid (aa) position of SARS-CoV-2 fragments, showing a heat map of antibody levels in each group for overlapping regions of different aa length. Proteins are indicated outside the circle plot followed by a line graph showing the sequence homology of other CoVs with SARS-CoV-2 for each gene. Proteins and protein fragments produced *in vitro* are indicated by bars and show length and position of each fragment in each protein. Each fragment is drawn twice and shows group mean normalized $\log_2$ signal intensity of antibody binding to each fragment for COVID-19 patient samples (P) and negative control sera (N). The purified receptor binding domain (RBD) is additionally shown for comparison. Signal intensity is shown by color gradients: IgG (grey to blue), IgA (grey to red), and IgM (grey to green). Bar pairs shown with gold outline represent significantly differential antibody binding between COVID-19 patients and healthy controls, defined as a mean $\log_2$ signal intensity ≥ 0.1 in at least one group and a t-test p value ≤ 0.05. The regions of greatest reactivity for each protein are outlined in magenta. Some fragments in E and M proteins that meet the reactivity threshold (grey) and are better visualized by individual responses as shown in Fig. S3. The Pearson's correlation coefficients ("Rho") between each full-length protein for each isotype are shown as links between protein sectors in the center of the circle (IgG: solid links, IgA: dashed, IgM: dotted). **(B)** A slice of the circular graphic is amplified and labeled in more detail as a guide to interpreting the full figure. The first 180 aa sequence of S2 is shown for IgG only.

Figure S2

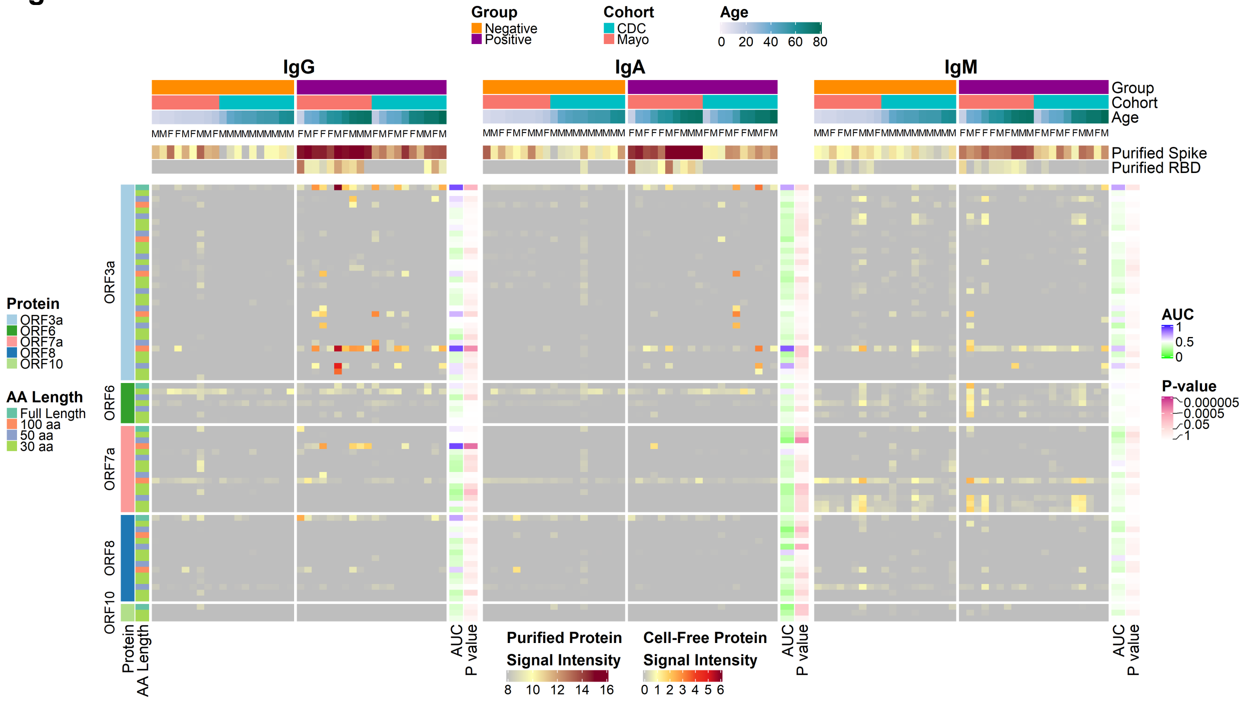

Figure S2. Reactivity in COVID-19 patient and healthy donor IgG (left heatmap), IgA (middle heatmap) and IgM (right heatmap) to SARS-CoV-2 nonstructural proteins and protein fragments produced *in vitro*. The heatmaps present the signals of individual array spots for the nonstructural SARS-CoV-2 proteins produced by IVTT. Columns represent serum samples, and rows represent IVTT proteins and protein fragments (n=34 ORF3a, n=7 ORF6, n=15 ORF7a, n=15 ORF8, n=3 ORF10). Antibody signal intensity to cell-free expressed proteins and fragments is shown on a color scale from grey to red. Sample information is overlaid above the heatmaps and includes sex (M/F), group (Negative or Positive), cohort (CDC or Mayo) and age (years), as well as the responses to purified S and RBD proteins on a separate color scale. Protein/fragment information is annotated to the left of the heatmaps and includes the full-length protein name and the amino acid length of the protein fragments (“AA Length”, as full length, 100, 50 or 30 aa). Proteins are ordered by the starting amino acid position for each fragment. For each isotype, the receiver operating characteristic area under the curve (AUC) and the unadjusted t-test *p* value between negatives and positives are shown to the right of each heatmap.

Figure S3

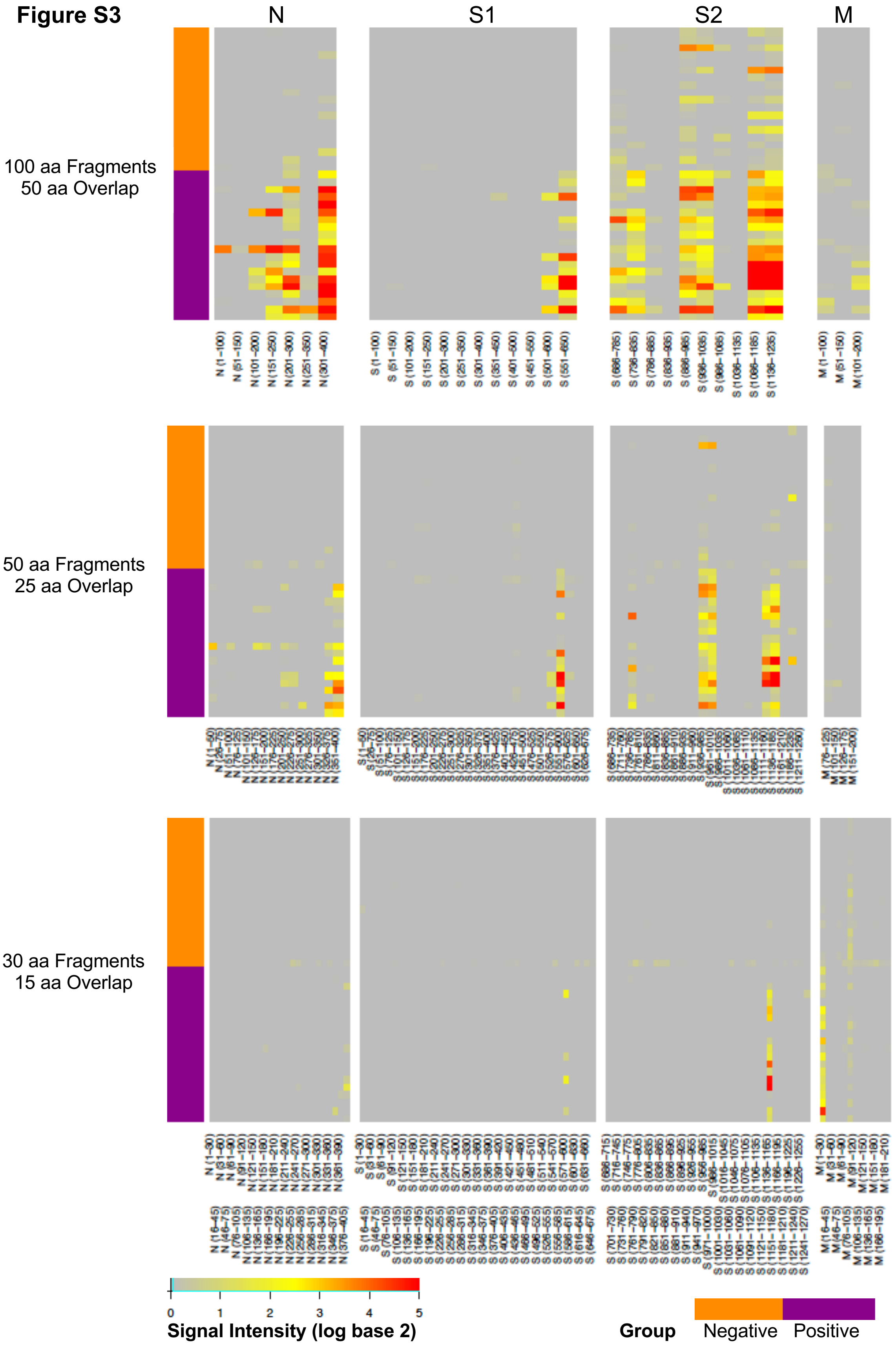

N
S1
S2
M
100 aa Fragments
50 aa Overlap
50 aa Fragments
25 aa Overlap
30 aa Fragments
15 aa Overlap
0
1
2
3
4
5
Signal Intensity (log base 2)
Group
Negative
Positive

Figure S3. COVID-19 patient IgG reactivity with SARS-CoV-2 N, S and M protein fragments arranged in amino acid sequence order. Heat maps show the IgG reactivity of each serum sample separately in each row. Columns denote each protein fragment as labeled; the 30 aa fragment labels are staggered to save space. A scale shows the meaning of the colors. Bars at left of each heat map identify the samples: orange indicates negative control samples and purple shows COVID-19 positive samples. Amino acid numbers indicate the positions of the fragments in each protein.

Figure S4

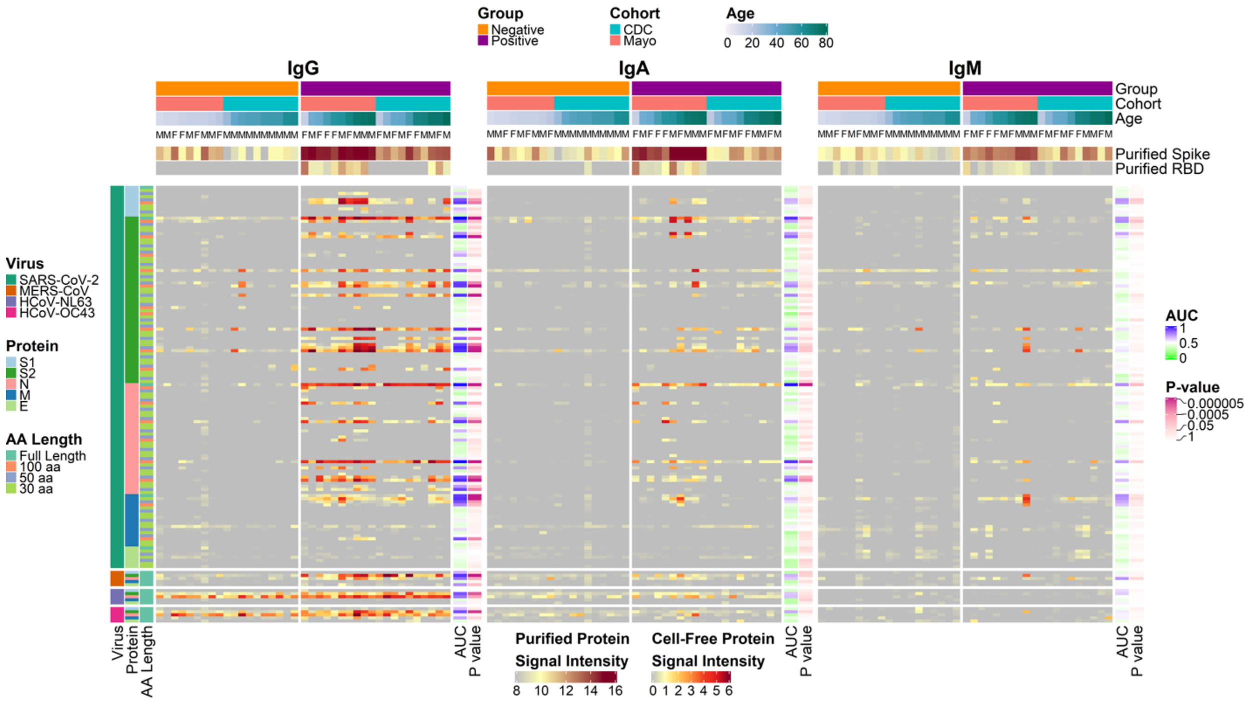

Figure S4. Reactivity in COVID-19 positive and negative IgG (left heatmap), IgA (middle heatmap) and IgM (right heatmap) to SARS-CoV-2 and other HCoV proteins and protein fragments produced *in vitro*. The heatmaps present the signals of antibody binding to individual proteins and protein fragments within the antigenic regions of SARS-CoV-2, as well as the full-length structural proteins of MERS-CoV, HCoV-NL63 and HCoV-OC43, for individual samples. Columns represent serum samples ordered by increasing age within group and cohort, and rows represent proteins or protein fragments; 128 SARS-CoV-2 proteins or fragments, five proteins each of MERS-CoV, HCoV-OC43 and HCoV-NL63. Antibody signal intensity to cell-free expressed proteins and fragments is shown on a color scale from grey to red. Sample information is overlaid above the heatmaps and includes sex (M/F), group (Negative or Positive), cohort (CDC or Mayo) and age (years), as well as the responses to purified S and RBD proteins on a separate color scale. Protein/fragment information is annotated to the left of the heatmaps and includes the virus, full-length protein name and the amino acid length of the protein fragments (“AA Length”, as full length, 100, 50 or 30 aa). For each isotype, the receiver operating characteristic area under the curve (AUC) and the unadjusted t-test *p* value between negatives and positives are shown to the right of each heatmap.

Figure S5

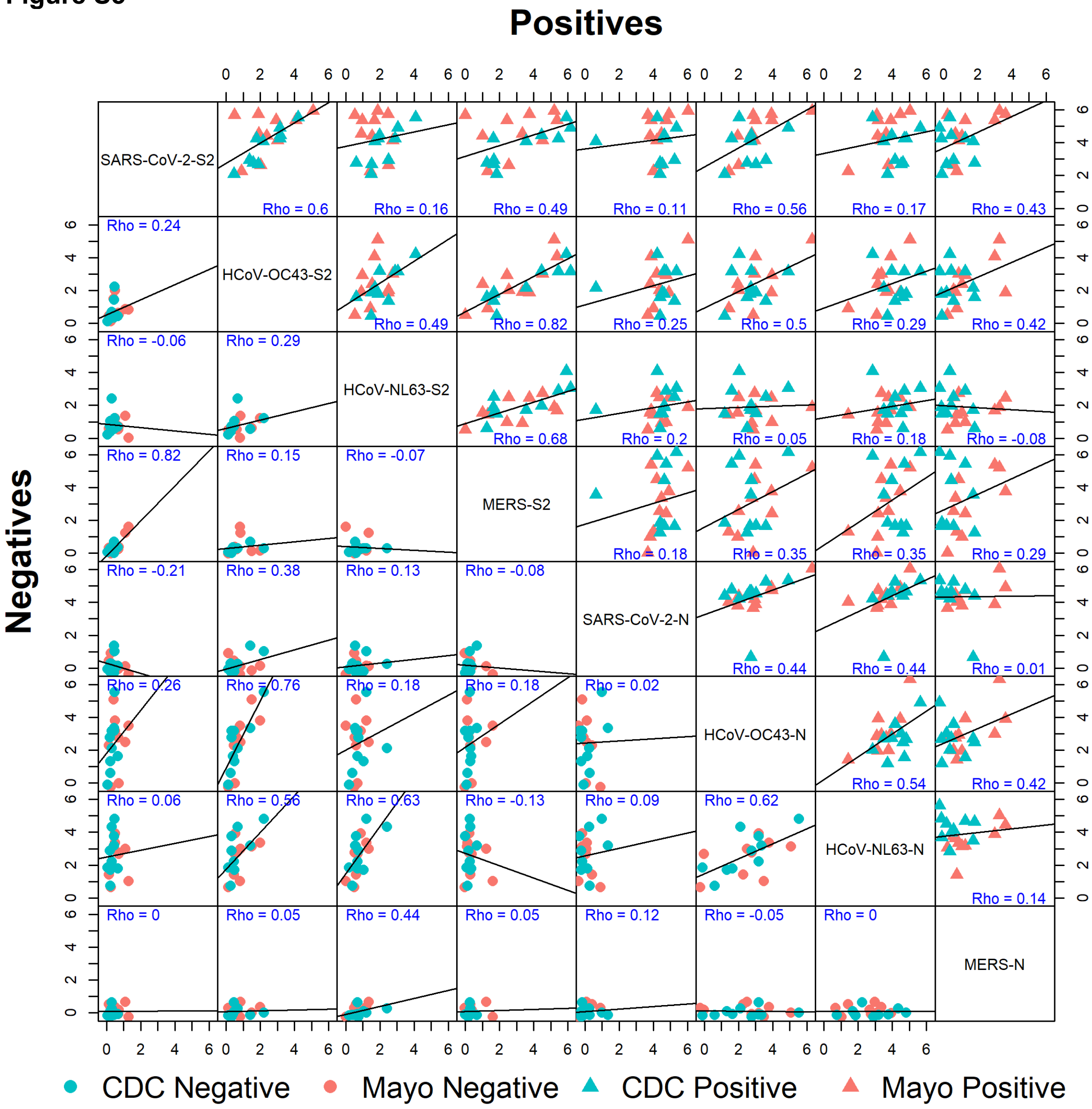

Figure S5. Correlation between IgG responses to SARS-CoV-2 and other human coronavirus N and S2 proteins. The correlation matrix shows the Pearson's correlation coefficient ("Rho", blue) between IgG normalized signal intensity to SARS-CoV-2, MERS-CoV, HCoV-OC43 and HCoV-NL63 N and S2 full-length proteins produced *in vitro*. The lower half of the diagonal shows correlation between proteins in the negative group (circles), and the upper half of the diagonal shows the positive group correlations (triangles). Rho values and a linear regression line are overlaid on each comparison.

Figure S6

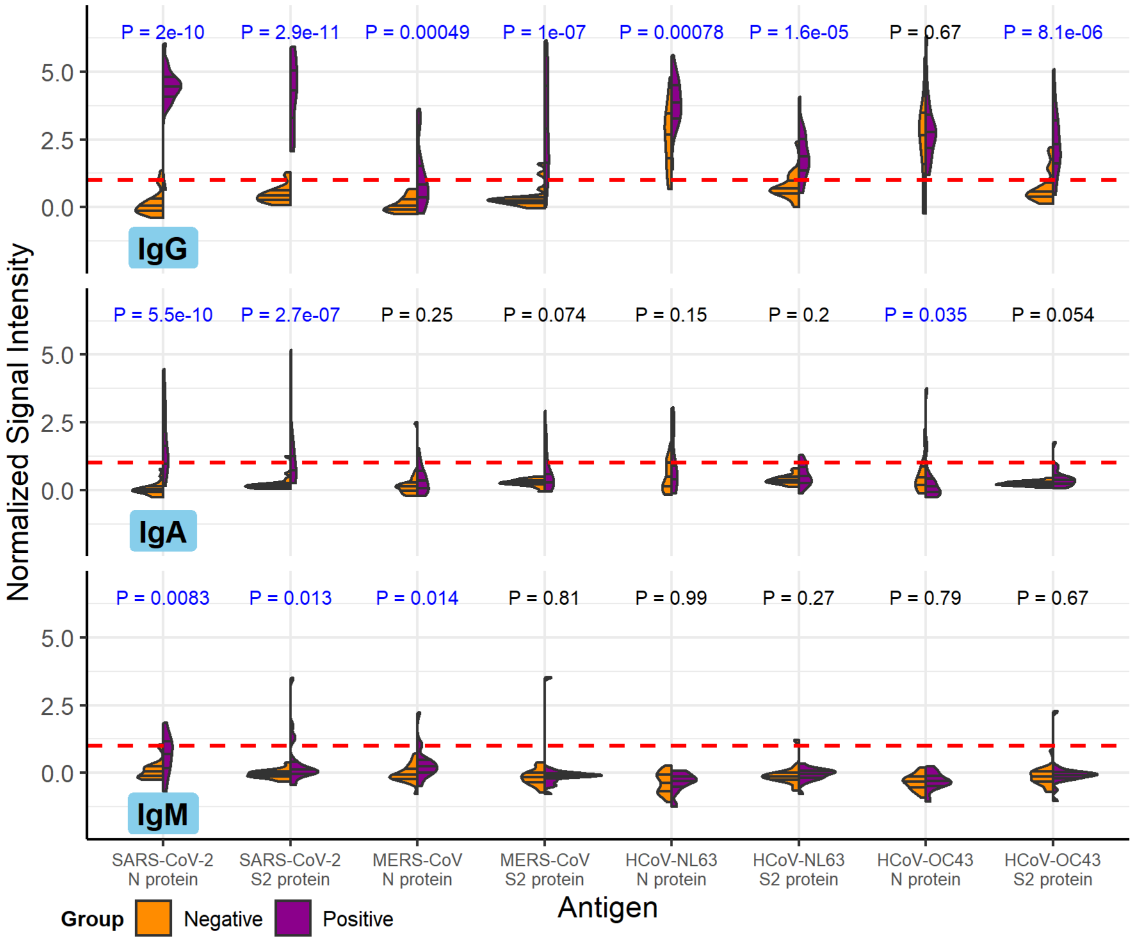

Figure S6. Differential reactivity between IgG, IgA and IgM responses to SARS-CoV-2 and other human coronavirus N and S2 proteins. The results show the differential reactivity between IgG, IgA and IgM normalized signal intensity to SARS-CoV-2, MERS-CoV, HCoV-OC43 and HCoV-NL63 N and S2 full-length proteins produced *in vitro* on a log scale, base 2. The split violin plot shows the normalized signal intensity distribution of each of the IVTT N and S2 proteins. Within each half-violin are three lines representing the interquartile range and the median. Above each split violin is the Wilcoxon rank sum *p* value, colored blue for significant *p* values below 0.05. The red dashed line represents the 1.0 seropositivity cutoff; values below the line represent non-specific binding to the *E. coli* expression system. The healthy control negative group is shown on the left violin halves in orange, and the COVID-19 patient positive group is shown in the right violin halves in purple.
